# Usefulness of plasminogen activator inhibitor-1 as a predictive marker of mortality in sepsis

**DOI:** 10.1186/s40560-017-0238-8

**Published:** 2017-07-11

**Authors:** Kota Hoshino, Taisuke Kitamura, Yoshihiko Nakamura, Yuhei Irie, Norihiko Matsumoto, Yasumasa Kawano, Hiroyasu Ishikura

**Affiliations:** Department of Emergency and Critical Care Medicine, Faculty of Medicine, Fukuoka University, 7-45-1 Nanakuma, Jonan-ku, Fukuoka 814-0180 Japan

**Keywords:** Disseminated intravascular coagulation, Fibrinolysis, Pathogen-associated molecular patterns, Sepsis-3

## Abstract

**Background:**

Sepsis is one of the most significant causes of mortality in intensive care units. It indicates crosstalk between inflammation and coagulation. In this study, we aimed to identify prognostic markers among sepsis biomarkers and coagulation/fibrinolysis markers.

**Methods:**

Patients with sepsis according to the Sepsis-3 criteria were enrolled from January 2013 to September 2015. Univariate and multivariate logistic regression analyses were performed to identify an independent predictive marker of 28-day mortality among sepsis biomarkers and coagulation/fibrinolysis markers on ICU admission. Receiver operating characteristic analysis was performed; the optimal cutoff value of 28-day mortality was calculated using the predictive marker. Patients were classified into two groups according to the cutoff level of the predictive marker. Patient characteristics were compared between the groups.

**Results:**

A total of 186 patients were enrolled in this study; the 28-day mortality was 19.4% (36/186). PAI-1 was identified as the only independent predictive marker of 28-day mortality by univariate and multivariate logistic regression. The area under the curve was 0.72; the optimal cutoff level was 83 ng/ml (sensitivity, 75%; specificity, 61%). Patients were classified into a higher group (PAI-1 level ≥83 ng/ml; *n* = 85) and a lower group (PAI-1 level <83 ng/ml; *n* = 101). All disseminated intravascular coagulation (DIC) scores and Sequential Organ Failure Assessment score were significantly higher in the higher group than in the lower group.

**Conclusions:**

PAI-1 can predict prognosis in sepsis patients. PAI-1 reflects DIC with suppressed fibrinolysis and organ failure, with microthrombi leading to microcirculatory dysfunction.

## Background

Sepsis is one of the most significant causes of mortality in intensive care units [1], and mortality among septic shock patients has been reported to be 30–50% [2, 3]. To improve the prognosis of sepsis patients, it is important to diagnose and immediately treat sepsis. The usefulness of sepsis biomarkers, such as procalcitonin (PCT) and presepsin (PSEP), has been reported; PCT and PSEP have been reported to be superior to C-reactive protein (CRP) and interleukin-6 for sepsis diagnosis and assessment of sepsis severity [4–6].

The defensive role of thrombosis is referred to as immunothrombosis [7]. Immunothrombosis designates an innate immune response induced by the formation of thrombi in microvessels. However, if left uncontrolled, immunothrombosis can eventually lead to disseminated intravascular coagulation (DIC) [8, 9]. Previous studies reported that the frequency of DIC in sepsis patients was 20–40% [10–13]. Therefore, it is important to measure coagulation/fibrinolysis markers as well as sepsis biomarkers in sepsis to assess the presence of crosstalk between inflammation and coagulation.

In this study, we aimed to identify prognostic markers among sepsis biomarkers and coagulation/fibrinolysis markers.

## Methods

### Patient selection

**Fig. 1:**
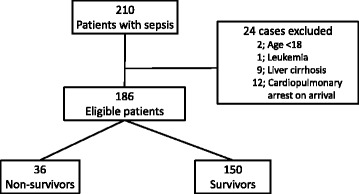
Flow chart. Flow diagram of patients who met the inclusion/exclusion criteria for the study population

This retrospective single-center study was approved by the ethics committee of Fukuoka University Hospital (No. 16-3-14). The criteria for admission to the ICU in patients with sepsis include one or more organ failures including shock or disturbance of consciousness. Patients with sepsis were enrolled from January 2013 to September 2015. The diagnosis of sepsis was based on the definition of Sepsis-3 [14]. The exclusion criteria were age <18 years, presence of leukemia, liver cirrhosis, and cardiopulmonary arrest on admission and occurrence of death within 24 h of ICU admission. Patients were classified into non-survivor and survivor groups on day 28 of ICU admission (Fig. 1).

### Patient characteristics

The two groups of patients were compared in terms of age, sex, infection focus, vital signs, the Japanese Association for Acute Medicine DIC score [15] along with the positive rate, the Sequential Organ Failure Assessment (SOFA) score [16] on ICU admission, and therapeutic agents. Moreover, sepsis biomarkers and coagulation/fibrinolysis markers were compared between non-survivor and survivor groups.

### Identification of predictive markers

In this study, we examined sepsis biomarkers and coagulation/fibrinolysis markers in blood samples on ICU admission. First, univariate logistic regression analyses were performed. The explanatory variables were CRP, PCT, and PSEP as sepsis biomarkers and platelet counts, prothrombin time international normalized ratio (PT-INR), activated partial thromboplastin time, antithrombin, D-dimer, thrombin-antithrombin complex (TAT), plasmin-α2 plasmin inhibitor complex, protein C (PC), thrombomodulin (TM), soluble fibrin (SF), and plasminogen activator inhibitor-1 (PAI-1) as coagulation/fibrinolysis markers. The response variable was 28-day mortality. Subsequently, multivariate logistic regression analysis was performed to identify the independent predictive marker of 28-day mortality using the markers that were identified as significant in univariate logistic regression.

### Cutoff value of the predictive marker and relationship with each score

Receiver operating characteristic (ROC) analysis was performed and the optimal cutoff value of 28-day mortality was calculated using the marker that was selected in multivariate logistic regression.

### Relationship between the predictive marker and sepsis severity

We divided patients into the following two groups considering the optimal cutoff value: the higher group and the lower group. Patient characteristics were compared between these two groups. The correlations of the predictive marker with DIC and SOFA scores were examined to evaluate the relationship between the predictive marker and each score. In addition, the 28-day survival rate was compared between the higher and the lower groups using the Kaplan–Meier analysis.

### Time course of sepsis biomarkers and coagulation/fibrinolysis markers

Time course of sepsis biomarkers and coagulation/fibrinolysis markers that were significantly different according to the univariate analyses were compared between the non-survivor and survivor groups.

### Assay of sepsis biomarkers and coagulation/fibrinolysis markers

Blood samples were routinely collected for measuring markers, and there were no lack of data on ICU admission in this study. CRP levels were measured by CRP-LATEX (II) *X*2 “SEIKEN” (Denka Seiken Co., Ltd, Tokyo, Japan) using EDTA plasma as a sample. PCT levels were measured by the Elecsys BRAHMS PCT assay (Roche Diagnostics, Tokyo, Japan) using EDTA plasma as a sample. PSEP levels were measured using a compact-automated immunoanalyzer, PATHFAST, based on a chemiluminescent enzyme immunoassay (CLEIA) (Mitsubishi Chemical Medience Corp., Japan). Platelet counts were measured in whole blood using an XT-1800i (Sysmex Co., Kobe, Japan). PT, APTT, AT, D-dimer, PIC, PC, and SF levels were measured in the plasma using a Coapresta 2000 (Sekisui Medicak, Tokyo, Japan). TAT, TM, and PAI-1 levels were measured using a STACIA (Mitsubishi Chemical Medience Corp., Tokyo, Japan). Total PAI-1 including active PAI-1 and tPA-PAI-1 complex was defined as PAI-1 in this study.

### Statistical analysis

Continuous variables are presented as median (interquartile range). Comparisons between groups were performed using the chi-square test for dichotomous variables and Mann–Whitney *U* test for continuous variables. ROC analysis, including determination of the area under the curve (AUC), was performed to determine the significance of the marker level for predicting 28-day mortality. The Youden index was used to identify the cutoff value. Correlations between the predictive marker and each score were evaluated using Spearman’s rank test. A *P* value of <0.05 was considered statistically significant. All statistical analyses were performed using JMP version 12 (SAS institute Japan, Tokyo, Japan).

## Results

### Patient selection

Patient enrollment into the study and exclusion from the study are shown in Fig. 1. Twenty-four of the 210 patients were excluded according to exclusion criteria. A total of 186 patients were enrolled in this study, and the 28-day mortality rate was 19.4% (36/186). Of the 186 patients, 36 patients were classified in the non-survivor group and 150 in the survivor group.

### Patient characteristics

Patient characteristics are presented in Table 1. There were significant differences in age, infection focus, SOFA score (in particular, cardiovascular and renal SOFA scores), and continuous renal replacement therapy; however, there was no significant difference among other patient characteristics. With respect to the comparison of sepsis biomarkers and coagulation/fibrinolysis markers, PCT, PT-INR, APTT, TAT, SF, and PAI-1 levels were significantly higher in the non-survivor group than in the survivor group. On the other hand, AT and PC levels were significantly lower in the non-survivor group than in the survivor group (Table 2).

**Table 1:** Comparison of patient characteristics

	Total *n* = 186	Non-survivors *n* = 36	Survivors *n* = 150	*P* value
Age (years old)	72 (62–79)	76 (68–81)	71 (61–78)	<0.05
Male	115 (62)	20 (56)	95 (63)	0.39
Infection focus				<0.05
Lung	77 (41)	8 (22)	69 (46)	
Abdomen	61 (33)	21 (58)	40 (27)	
Skin and soft tissue	13 (7)	3 (8)	10 (7)	
Urinary tract	11 (6)	2 (6)	9 (6)	
Others	11 (6)	1 (3)	10 (7)	
Unknown	13 (7)	1 (3)	12 (8)	
Mean blood pressure (mmHg)	78 (67–97)	74 (63–92)	79 (67–97)	0.13
Heart rate (bpm)	110 (95–120)	111 (95–120)	110 (95–121)	0.77
Respiratory rate (bpm)	23 (19–28)	24 (20–28)	22 (18–28)	0.28
Body temperature (°C)	36.9 (36.3-37.9)	36.7 (36.0-37.4)	36.9 (36.4-38.1)	0.06
JAAM DIC score	3 (2–5)	4 (2–6)	3 (2–5)	0.13
JAAM DIC positive rate	87 (47)	21 (58)	66 (44)	0.12
SOFA score	8 (5–11)	11 (8–13)	8 (5–11)	<0.01
Respiratory	2 (2–3)	2 (1–3)	2 (2–3)	0.87
Cardiovascular	1 (0–4)	4 (0–4)	1 (0–4)	<0.01
Liver	0 (0–1)	0 (0–1)	0 (0–1)	0.95
Renal	1 (0–3)	3 (1–4)	1 (0–3)	<0.01
Coagulation	1 (0–2)	0 (0–2)	1 (0–2)	0.85
CNS	1 (0–2)	1 (0–3)	1 (0–2)	0.95
rhs TM	46 (25)	11 (31)	35 (23)	0.37
AT III	28 (15)	6 (17)	22 (15)	0.76
IVIG	92 (49)	21 (58)	71 (47)	0.24
CRRT	44 (24)	17 (47)	27 (18)	<0.01
PMX-DHP	28 (15)	9 (25)	19 (13)	0.06

Data are presented as median (interquartile range) or number (percentage)

*JAAM* Japanese Association for Acute Medicine, *DIC* disseminated intravascular coagulation, *SOFA* sequential organ failure assessment, *CNS* central nervous system, rhs TM recombinant human soluble thrombomodulin, *AT* antithrombin, *IVIG* intravenous immunoglobulin, *CRRT* continuous renal replacement therapy, *PMX-DHP* direct hemoperfusion with polymyxin B-immobilized fiber

**Table 2:** Comparison of sepsis biomarkers and coagulation/fibrinolysis markers

	Total *n* = 186	Non-survivors *n* = 36	Survivors *n* = 150	*P* value
CRP (mg/dl)	12 (5–20)	15 (9–21)	12 (4–20)	0.21
PCT (ng/ml)	7 (1–42)	18 (2–89)	6 (1–33)	<0.05
PSEP (pg/ml)	839 (440–1597)	905 (546–1811)	815 (422–1562)	0.12
Platelet count (×104/μl)	14.8 (8.6–23.0)	16.6 (5.8–25.3)	14.4 (9.0–22.3)	0.81
PT-INR	1.3 (1.2–1.6)	1.5 (1.3–2.1)	1.3 (1.2–1.5)	<0.05
APTT (s)	35 (30–42)	39 (33–45)	34 (30–41)	<0.05
AT (%)	70 (56–87)	63 (48–77)	73 (58–87)	<0.05
D-dimer (μg/ml)	6.8 (2.9–15.7)	8.4 (3.3–18.2)	6.7 (2.8–14.6)	0.27
TAT (ng/ml)	6.9 (3.7–16.5)	8.9 (5.5–34.8)	6.5 (3.2–13.0)	<0.05
PIC (μg/ml)	1.7 (1.0–3.3)	1.5 (0.8–3.2)	1.7 (1.1–3.4)	0.19
PC (%)	47 (33–70)	38 (26–56)	52 (34–70)	<0.01
TM (U/ml)	33 (23–47)	40 (25–52)	32 (23–45)	0.12
SF (μg/ml)	21 (12–62)	45 (17–80)	20 (12–43)	<0.01
PAI-1 (ng/ml)	66 (29–191)	154 (73–527)	51 (26–154)	<0.01

Data are presented as median (interquartile range)

*CRP* C-reactive protein, *PCT* procalcitonin, *PSEP* presepsin, *PT-INR* prothrombin time-international normalized ratio, *APTT* activated partial thromboplastin time, *AT* antithrombin, *TAT* thrombin-antithrombin complex, *PIC* plasmin α2-plasmin inhibitor complex, *PC* protein C, *TM* thrombomodulin, *SF* soluble fibrin, *PAI-1* plasminogen activator inhibitor-1

### Identification of predictive markers

PSEP (*P* < 0.05) as a sepsis biomarker, TAT (*P* < 0.05), PC (*P* < 0.01), SF (*P* < 0.01), and PAI-1 (*P* < 0.01) as coagulation/fibrinolysis markers, and SOFA score (*P* < 0.01) were selected in univariate logistic regression (Table 3). Subsequently, multivariate logistic regression was performed using PSEP, TAT, PC, SF, PAI-1, and SOFA score as explanatory variables. PAI-1 was found to be the only independent predictive marker of 28-day mortality (*P* < 0.05; Table 3).

**Table 3:** Logistic regression analyses of the coagulation/fibrinolysis markers for 28-day mortality

	Univariate analyses			Multivariate analyses		
Markers	OR	95% CI	*P* value	OR	95% CI	*P* value
CRP (mg/dl)	1.020	0.986–1.054	NS			
PCT (ng/ml)	1.000	0.993–1.003	NS			
PSEP (pg/ml)	1.000	1.000–1.000	<0.05	1.000	0.999–1.000	NS
Platelet count (×10^4^/μl)	1.003	0.973–1.028	NS			
PT-INR	0.993	0.834–1.034	NS			
APTT (s)	1.013	0.992–1.033	NS			
AT (%)	0.983	0.966–1.000	NS			
D-dimer (μg/ml)	1.005	0.995–1.015	NS			
TAT (ng/ml)	1.015	1.003–1.027	<0.05	0.999	0.982–1.014	NS
PIC (μg/ml)	1.021	0.940–1.094	NS			
PC (%)	0.977	0.959–0.993	<0.01	0.986	0.967–1.004	NS
TM (U/ml)	1.005	0.996–1.014	NS			
SF (μg/ml)	1.021	1.008–1.034	<0.01	1.014	0.997–1.031	NS
PAI-1 (ng/ml)	1.002	1.001–1.003	<0.01	1.002	1.000–1.003	<0.05
SOFA score	1.200	1.078–1.348	<0.01	1.075	0.943–1.231	NS

*OR* odds ratio, *CI* confidence interval, *CRP* C-reactive protein, *PCT* procalcitonin, *PSEP* presepsin, *PT-INR* prothrombin time-international normalized ratio, *APTT* activated partial thromboplastin time, *AT* antithrombin, *TAT* thrombin-antithrombin complex, *PIC* plasmin α2-plasmin inhibitor complex, *PC* protein C, *TM* thrombomodulin, *SF* soluble fibrin, *PAI-1* plasminogen activator inhibitor-1, *SOFA* sequential organ failure assessment

### Cutoff value of the predictive marker and relationship with each score

With regard to the accuracy of predicting 28-day mortality based on the level of PAI-1 in the ROC analysis, the AUC was 0.72 and the optimal cutoff value was 83 ng/ml (sensitivity, 75%; specificity, 61%).

### Relationship between the predictive marker and sepsis severity

**Fig. 2:**
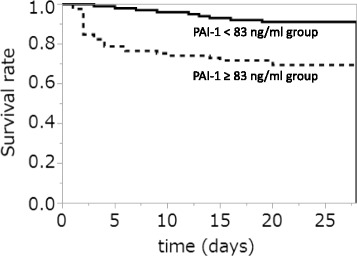
Kaplan–Meier analysis. The survival rate is significantly higher in the higher group (PAI-1 level ≥83 ng/ml; *n* = 85) than that in the lower group (PAI-1 level <83 ng/ml; *n* = 101) (log-rank test, *P* < 0.01)

Patients were classified into the higher group (PAI-1 level ≥83 ng/ml; *n* = 85) and the lower group (PAI-1 level <83 ng/ml; *n* = 101). DIC, SOFA scores (in particular, cardiovascular and coagulation SOFA scores), and 28-day mortality were significantly higher in the higher group than those in the lower group (all *P* < 0.01; Table 4). To determine which scores affect the PAI-1 level the most, correlations between the PAI-1 level and DIC or SOFA score were evaluated using Spearman’s rank test. We noted positive correlations between the PAI-1 level and DIC score (*r* = 0.18, *P* < 0.05) or SOFA score (*r* = 0.32, *P* < 0.01), in particular, cardiovascular (*r* = 0.35, *P* < 0.01) and renal (*r* = 0.22, *P* < 0.01; Table 5). The 28-day survival rate according to Kaplan–Meier analysis was significantly lower in the higher group than that in the lower group (log-rank test, *P* < 0.01; Fig. 2).

**Table 4:** Patient characteristics of the PAI-1 higher and lower groups

	PAI-1 ≥83 ng/ml *n* = 85	PAI-1 <83 ng/ml *n* = 101	*P* value
Age	74 (63–80)	71 (61–78)	0.14
Male	50 (59)	65 (64)	0.44
JAAM DIC score	4 (2–6)	2 (1–5)	<0.01
JAAM DIC positive rate	52 (61)	35 (35)	<0.01
SOFA score	10 (8–12)	7 (4–10)	<0.01
Respiratory	2 (2–3)	2 (1–3)	0.42
Cardiovascular	4 (0–4)	0 (0–3)	<0.01
Liver	0 (0–1)	0 (0–0)	0.09
Renal	2 (0–3)	1 (0–3)	0.18
Coagulation	1 (0–2)	0 (0–1)	<0.01
CNS	1 (1–3)	1 (0–2)	0.12
28-day mortality	27 (32)	9 (9)	<0.01

Data are presented as median (interquartile range) or number (percentage)

*PAI-1* plasminogen activator inhibitor-1, *JAAM* Japanese Association for Acute Medicine, *DIC* disseminated intravascular coagulation, *SOFA* sequential organ failure assessment, *CNS* central nervous system

**Table 5:** Correlations between PAI-1 and each score

	Correlation coefficient	*P* value
JAAM DIC score	0.18	<0.05
SOFA score	0.32	<0.01
Respiratory	−0.01	0.90
Cardiovascular	0.35	<0.01
Liver	0.06	0.40
Renal	0.22	<0.01
Coagulation	0.13	0.08
CNS	0.13	0.08

*PAI-1* plasminogen activator inhibitor-1, *JAAM* Japanese Association for Acute Medicine, *DIC* disseminated intravascular coagulation, *SOFA* sequential organ failure assessment, *CNS* central nervous system

### Time course of sepsis biomarkers and coagulation/fibrinolysis markers

**Fig. 3:**
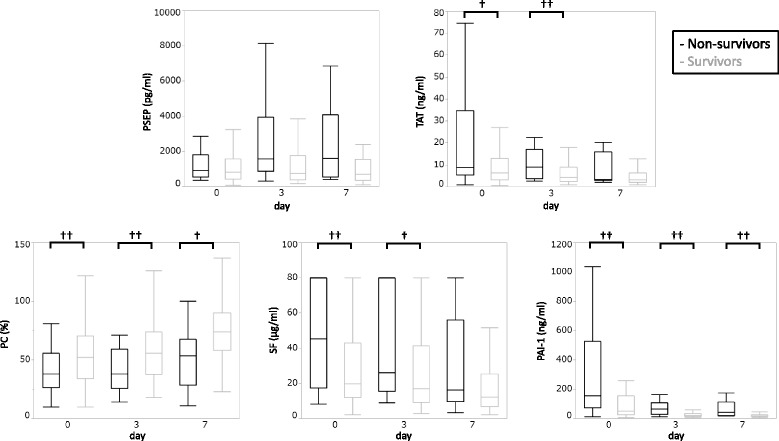
Time courses of sepsis biomarker and coagulation/fibrinolysis marker levels. This figure shows time course (days 0, 3, and 7) of PSEP, TAT, PC, SF, and PAI-1 levels that were significantly different according to univariate analyses. †*P* < 0.05, ††P < 0.01 according to Mann–Whitney *U* test

Figure 3 shows the time courses of PSEP, TAT, PC SF, and PAI-1 that were significantly different according to univariate analyses. PC and PAI-1 levels were significantly different between the non-survivor and survivor groups on days 0, 3, and 7 (*P* < 0.05). In particular, PAI-1 levels at all time courses were significantly higher in the non-survivor group than those in the survivor group (*P* < 0.01).

## Discussion

In this study, we used the new sepsis definition “Sepsis-3” and identified the predictive marker of 28-day mortality among sepsis biomarkers and coagulation/fibrinolysis markers. This study revealed that PAI-1 was the most predictive marker according to the multivariate analysis, and the cutoff value was 83 ng/ml. Morbidities of DIC and organ dysfunctions were significantly higher in the higher PAI-1 group. Moreover, PAI-1 was correlated with SOFA score, in particular cardiovascular and renal scores. PAI-1 levels were significantly higher in the non-survivor group than those in the survivor group during the time course as well as on admission.

The first definition of septic syndrome established in 1992 was based on the concomitant presence of presumed/confirmed infection and at least two of the four Systemic Inflammatory Response Syndrome criteria [17]. The new sepsis definition “Sepsis-3” mentions life-threatening organ dysfunction caused by a dysregulated host response to infection [14]. Therefore, it is important to assess organ dysfunction that is caused by coagulo-fibrinolytic abnormalities in sepsis. This is the first study to evaluate the prognostic value of sepsis biomarkers and coagulation/fibrinolysis markers using the Sepsis-3 definition.

There are some reports that sepsis biomarkers are useful for predicting mortality in patients with sepsis. Clec’h et al. [18] reported that PCT was able to predict mortality in patients with septic shock. Jensen et al. [19] reported that PCT level increase denoted a high risk of mortality. In the multicenter, retrospective, case–control study, PSEP was the only predictive marker of mortality according to multivariate analysis including PCT and PSEP [20]. In this study, PSEP was significant according to the univariate analysis; however, PSEP was not the predictive marker according to the multivariate analysis.

In sepsis, innate immune responses start following the identification of pathogen-associated molecular patterns or damage-associated molecular patterns by pattern-recognition receptors, such as Toll-like receptors expressed on immunocompetent cells and the endothelium. The sensed danger signals activate both intracellular signal transduction pathways and plasma cascades, which together produce pro-inflammatory cytokines, further stimulating the production of inflammatory biomarkers. The actions of inflammatory cytokines trigger the production of large amounts of tissue factor from monocytes/macrophages and the vascular endothelium, thus leading to marked coagulation activation. PAI-1 is synthesized by endothelial cells and hepatocytes. It is the main inhibitor of tissue-type plasminogen activator and plays an important role in the regulation of fibrinolysis. Elevated levels of PAI-1 result in deficient plasminogen activation and are a risk factor for thrombosis, including DIC. It is already known that PAI-1 level is markedly increased in sepsis, fibrinolysis is strongly suppressed, and dissolution of multiple microthrombi is more difficult [21], and because of microcirculatory impairment, severe organ dysfunction may occur [22, 23]. On the other hand, PAI-1 is not involved in malignant tumors such as acute leukemia and solid cancers [23].

Koyama et al. [24] reported that TAT, PC, and PAI-1 were predictive markers of mortality in patients with sepsis. TAT, PC, and PAI-1 were significant according to the univariate analysis as well as a previous study [24], and PAI-1 was finally the only predictive marker in this study. Madoiwa et al. [25] examined 117 patients with sepsis-induced DIC and reported that the hazard ratio of 28-day mortality was increasing 23 times with a PAI-1 level of >90 ng/ml. The 90 ng/ml PAI-1 level is similar to the PAI-1 cutoff value of 83 ng/ml in this study. A previous study [24] revealed that the PAI-1 cutoff value was 269 ng/ml; however, the PAI-1 cutoff value in this study was 83 ng/ml. There was a huge difference in the PAI-1 cutoff value between the two studies. Although there is no unified method for PAI-1 level measurement, there are a few reagents for this measurement. The difference in PAI-1 cutoff values may be affected by reagents.

When considering the PAI-1 cutoff level, DIC and SOFA scores were significantly higher in patients with a PAI-1 level of ≥83 ng/ml than in those with a PAI-1 level of <83 ng/ml (all *P* < 0.01). The 28-day survival rate was significantly lower in patients with a level of ≥83 ng/ml than in those with a PAI-1 level of <83 ng/ml (*P* < 0.01). These results suggest that patients with a PAI-1 level of ≥83 ng/ml tend to develop DIC with suppressed fibrinolysis and multiple organ dysfunction. Madoiwa et al. [25] reported that the PAI-1 level correlated with the SOFA score. The results of this previous study are compatible with our results that there was a positive correlation between PAI-1 and the SOFA score in sepsis patients (*r* = 0.32, *P* < 0.01).

Vincent et al. [26] studied the relationship of this dysfunction with the outcome. Increased cardiovascular, central nervous system, or renal SOFA score was related to high mortality. Furthermore, both cardiovascular and renal failures cause the highest mortality. In this study, cardiovascular and renal SOFA scores were significantly higher in the non-survivors’ group than in the survivors’ group. PAI-1 levels were significantly correlated with cardiovascular and renal scores in each SOFA scores. PAI-1 levels reflect the process that sepsis causes organ failures, resulting in death. Moreover, PAI-1 level is superior to SOFA score for predicting mortality.

Fibrinolysis is suppressed in sepsis and multiple microthrombi impair microcirculation, resulting in organ failure. The PAI-1 level reflects this process and can predict mortality in sepsis. In this study, sepsis was diagnosed according to the new definition of “Sepsis-3.” PAI-1 reflects coagulo-fibrinolytic abnormalities, particularly suppressed fibrinolysis and organ failure along with microthrombi, leading to microcirculatory dysfunction in sepsis. Therefore, PAI-1 may be a useful marker to assess sepsis severity according to the Sepsis-3 definition.

The present study has some limitations. This was a small, retrospective, single-center study. The sampling timing in the course of sepsis was not detected. Some patient characteristics between non-survivors and survivors were different; therefore, further studies are required to make sure of the efficacy of PAI-1. Additionally, only sepsis biomarkers and coagulation/fibrinolysis markers that could be examined in our center were used.

## Conclusion

PAI-1 can predict prognosis in sepsis patients. In addition, PAI-1 reflects DIC with suppressed fibrinolysis and organ failure, with microthrombi leading to microcirculatory dysfunction.
